# Multi-Source Remote Sensing Data for Wetland Information Extraction: A Case Study of the Nanweng River National Wetland Reserve

**DOI:** 10.3390/s24206664

**Published:** 2024-10-16

**Authors:** Hao Yu, Shicheng Li, Zhimin Liang, Shengnan Xu, Xin Yang, Xiaoyan Li

**Affiliations:** 1 Modern Industry College, Jilin Jianzhu University, Changchun 130118, China; lishicheng@student.jlju.edu.cn (S.L.); liangzhimin@student.jlju.edu.cn (Z.L.); yangxin@student.jlju.edu.cn (X.Y.); 2 Northeast Institute of Geography and Agroecology, Chinese Academy of Sciences, Changchun 130102, China; 3 Research Department, Chang Guang Satellite Technology Co., Ltd., Changchun 130102, China; 4 College of Earth Sciences, Jilin University, Changchun 130012, China; lxyan@jlu.edu.cn

**Keywords:** sentinel mission, multi-source data, pixel-based classification, object-based classification, land cover

## Abstract

Wetlands play a vital role in regulating the global carbon cycle, providing biodiversity, and reducing flood risks. These functions maintain ecological balance and ensure human well-being. Timely, accurate monitoring of wetlands is essential, not only for conservation efforts, but also for achieving Sustainable Development Goals (SDGs). In this study, we combined Sentinel-1/2 images, terrain data, and field observation data collected in 2020 to better understand wetland distribution. A total of 22 feature variables were extracted from multi-source data, including spectral bands, spectral indices (especially red edge indices), terrain features, and radar features. To avoid high correlations between variables and reduce data redundancy, we selected a subset of features based on recursive feature elimination (RFE) and Pearson correlation analysis methods. We adopted the random forest (RF) method to construct six wetland delineation schemes and incorporated multiple types of characteristic variables. These variables were based on remote sensing image pixels and objects. Combining red-edge features, terrain data, and radar data significantly improved the accuracy of land cover information extracted in low-mountain and hilly areas. Moreover, the accuracy of object-oriented schemes surpassed that of pixel-level methods when applied to wetland classification. Among the three pixel-based schemes, the addition of terrain and radar data increased the overall classification accuracy by 7.26%. In the object-based schemes, the inclusion of radar and terrain data improved classification accuracy by 4.34%. The object-based classification method achieved the best results for swamps, water bodies, and built-up land, with relative accuracies of 96.00%, 90.91%, and 96.67%, respectively. Even higher accuracies were observed in the pixel-based schemes for marshes, forests, and bare land, with relative accuracies of 98.67%, 97.53%, and 80.00%, respectively. This study’s methodology can provide valuable reference information for wetland data extraction research and can be applied to a wide range of future research studies.

## 1. Introduction

Wetlands are one of the most diverse ecosystems on Earth [1]. They play a crucial role in maintaining the water cycle, improving water quality, regulating floods, sequestering carbon, modulating climate, and conserving biodiversity [2,3,4]. Unfortunately, over the last 30 years, global warming, economic development, and intensive human activities have damaged or destroyed roughly two-thirds of the world’s wetlands [5]. Therefore, the ability to rapidly and accurately monitor and assess wetland size and stability is essential for protecting wetland resources and mitigating climate change.

Multi-source satellite data have been widely used for wetland detection [6]. The Landsat series is the most extensively applied due to its long temporal coverage [7]. However, the relatively low spatial (30 m) and temporal (8–16 d) resolution of Landsat data limits its applicability in high-precision wetland delineation [8]. Commercial satellites with high spatial resolution are costly, and their limited spectral bands are not suitable for large-scale wetland information extraction. Additionally, the applicability of optical satellites is restricted by their wavelength limitations, which prevents penetration through clouds [9]. In contrast, radar satellites operate day and night and can acquire imagery regardless of the weather [10,11]. Recently, the Sentinel-1 (radar) and Sentinel-2 (multispectral) Earth Observation Satellite missions have become available, providing remote sensing imagery with high spatial and temporal resolution, and are increasingly used in wetland detection studies [6,12]. Sentinel-1 performs C-band synthetic aperture radar imaging, offering dual polarization capability and rapid product delivery [13]. Its high spatial resolution and unique three vegetation red-edge bands facilitate precise land cover mapping [14,15,16]. Research has demonstrated that incorporating digital elevation model (DEM) data into wetland monitoring can enhance classification accuracy [15,17,18,19]. Consequently, this research focuses on leveraging multi-source remote sensing data, including optical, radar, and terrain datasets, to monitor wetlands with high precision and on a large scale.

Supervised machine learning methods that utilize remote sensing data, such as decision tree (DT), support vector machine (SVM), and convolutional neural networks (CNN), have been extensively employed for large-scale wetland mapping [20,21]. However, these approaches have limitations in completing multi-class classification tasks. For example, DTs tend to overfit and produce notably unstable noise data results [22,23]; SVMs are characterized by slow training time with extensive datasets and high memory usage, making them sensitive to tuning parameters [24,25,26]; CNNs, while powerful, are difficult to train due to their significant computational demands, and their “black box” nature compromises interpretability [27,28]. As a result, RF has emerged as the dominant tool for land cover identification due to its accuracy and efficiency [29]. Surpassing the predictive capabilities of individual decision trees, RF can accommodate large feature sets while still maintaining peak performance [29,30]. Hence, we employed RF in our study, aiming to elucidate its applicability over expansive regions.

Wetland information extraction has primarily relied on either pixel-based or object-based classification techniques. However, comparative analyses between these two methods remains woefully underexplored in existing literature. Pixel-based classification methods use remote sensing imagery to capture subtle variations and extract detailed wetland information from feature differences [31,32]. However, this meticulous extraction process often results in data redundancy, significantly impairing classification efficiency. Object-based image analysis (OBIA) divides satellite images into homogeneous objects, providing more useful results than individual pixels. OBIA can fully utilize spatial information (shape, terrain, etc.) and effectively reduce the “salt-and-pepper” effect of pixel classification [6,33]. However, the fine-tuning parameters of object-based techniques can be complex, requiring iterative optimization. Therefore, the aim of our research was to study wetland delineation using both pixel-based and object-based approaches, comparing their accuracy to evaluate the efficacy of these methods in wetland mapping.

This research utilized the Google Earth Engine (GEE) platform to obtain Sentinel-1/2 imagery and terrain data. We extracted and constructed spectral, terrain, and radar features to develop six experimental schemes based on both pixel and object methods. An analysis was conducted on the diverse response characteristics presented by wetlands, comparing the benefits of the various schemes employed in wetland delineation. Consequently, this research yielded the most effective classification approach, enabling highly precise extraction of land cover data for the Nanweng River National Wetland Reserve (NWR). The results could potentially provide data and a scientific basis for the management, protection, and sustainable development of NWR, while also offering methodological references for wetland research in other regions.

## 2. Materials

### 2.1. Overview of the Study Area

**Figure 1:**
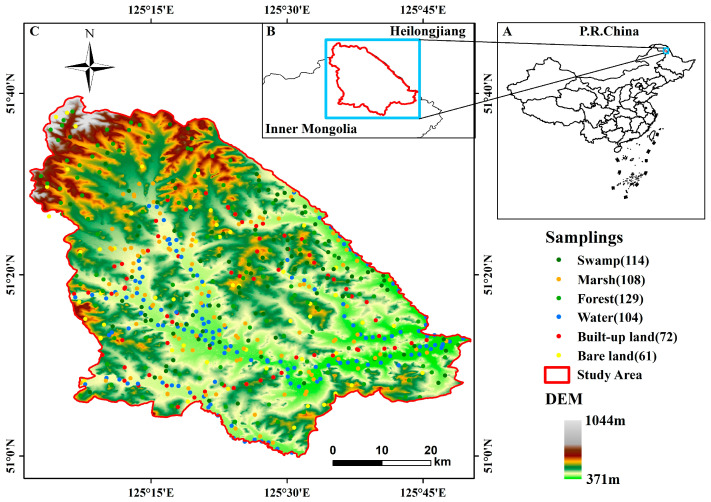
The topographical location of the study area with a distribution of the sample points.

NWR was established in the cold, temperate forest zone of Northeast China. It is located at the border of Heilongjiang Province and the Inner Mongolia Autonomous Region, between 50°59′–51°39′ N and 125°01′–125°50′ E (Figure 1). The terrain is characterized by higher elevations in the north and west and lower elevations in the south and east, with altitudes ranging from 370 to 1044 m above sea level [34]. The average annual temperature is −3 °C, and the annual rainfall is approximately 500 mm [35]. NWR is a significant part of the Nenjiang River system and is one of the largest forest–swamp areas in China, playing a crucial role in wetland ecological research and conservation.

### 2.2. Data Sources

#### 2.2.1. Remote Sensing Data and Pre-Processing

This study utilized Sentinel-1/2 satellite imagery with a spatial resolution of 10 m and a dual-satellite revisit frequency of 3–5 d [6]. Sentinel-1 employs a C-band synthetic aperture radar (SAR) and operates in interferometric wide (IW) mode [32]. Sentinel-2 carries an optical instrument payload that samples 13 spectral bands and is equipped with a multispectral sensor, allowing it to cover an array of wavelengths between visible light and shortwave infrared. We used Sentinel-1 Level-1 Ground Range Detected (GRD) SAR imagery and Sentinel-2 Multispectral Instrument (MSI) Level-2A (L2A) products, which provided atmospherically-corrected surface reflectance data [36,37]. The terrain data used was the 30-m resolution Digital Elevation Model jointly measured by NASA and the National Imagery and Mapping Agency (NIMA) (https://earthexplorer.usgs.gov/, accessed on 9 March 2024).

Data acquisition and preprocessing were conducted on the GEE platform. The Sentinel-2 imagery was selected to ensure cloud cover below 5%. Additionally, imaging dates were chosen between July and August, a period characterized by active vegetation growth and the absence of snow cover. The primary image used in this study was acquired on 19 August 2020. It was selected for its minimal cloud cover and underwent cloud removal and gap-filling using cloud-free images from nearby dates. Additionally, all spectral bands were upsampled to 10 m for calculating the spectral index and examine the diverse spectral characteristics. Radar data from 16 August 2020 were preprocessed, including orbit file correction, thermal noise removal, filtering, radiometric calibration, and Doppler terrain correction. The above data were stitched and clipped according to the NWR boundaries.

#### 2.2.2. Sample Data

Based on the land cover types in the NWR, as determined by the national classification standards, the land categories were divided into six types: swamp, marsh, forest, water, built-up land, and bare land. The sample data consisted of two primary components: randomly generated sample points and field sample data. The random sample points were automatically generated using GEE code, while the field sample data were collected through a series of field surveys using a portable BeiDou Navigation Satellite System (BDS) and an unmanned aerial vehicle (UAV). All sample points were quantitatively analyzed using the Jeffreys–Matusita (JM) distance algorithm, and 588 satisfactory sample points were selected [38]. A stratified random sampling method was employed, with 70% samples used for training and 30% for validation. This ensured that the same ratio of samples participated in both training and validation. The number of samples for each land category is shown in Figure 1.

#### 2.2.3. Constructing Characterization Variables

We selected and constructed spectral features, vegetation indices [39,40], water indices [41], red-edge indices [42,43,44,45], terrain features, and radar features on the GEE platform [5,46]. The spectral features included common visible bands, near-infrared bands, and shortwave infrared bands, as well as the three unique vegetation red-edge bands from Sentinel-2 (Supplementary Materials). The spectral indices included six red-edge indices and two non-red-edge indices (NDVI and NDWI), while the terrain features included DEM and slope. The radar features were the backscatter coefficients of VV and VH polarizations, providing additional information on soil moisture and surface roughness [47]. Specific details are provided in Table 1.

## 3. Methods

### 3.1. Overall Philosophy

**Figure 2:**
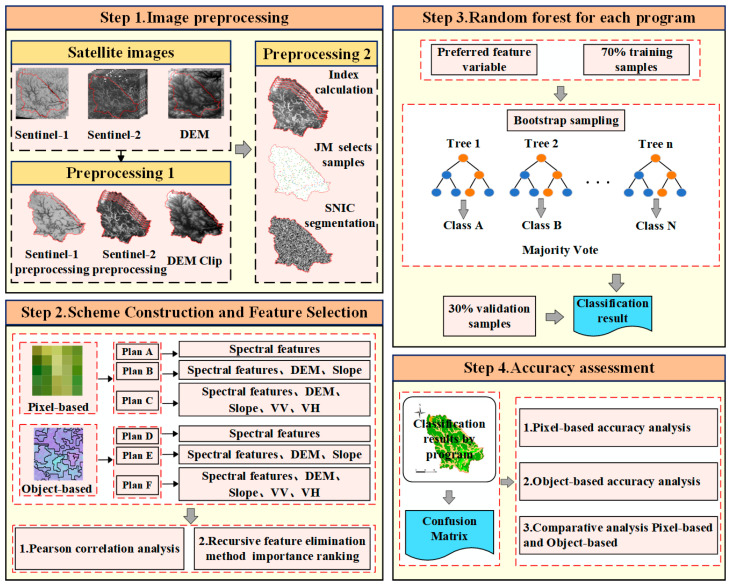
General workflow for wetland detection (SNIC is short for simple non-iterative clustering).

The aim of our study was to achieve high-precision extraction of land cover information in the wetland reserve. Our specific workflow is illustrated in Figure 2 and includes the following processes: (1) an in-depth analysis of the spectral characteristics, terrain features, and radar features of various land cover types; (2) the establishment of information extraction schemes, based on both pixel and object modes, initially utilizing only spectral bands and indices and subsequently integrating terrain features and radar features (Pearson correlation analysis and recursive feature elimination (RFE) were used to select the feature variables for each scheme, and RF was employed to perform land cover information extraction in both the pixel and object modes); and (3) a comparative analysis of the advantages of different schemes in extracting various land cover types. Detailed information about the schemes is provided in Table 2.

### 3.2. Object-Oriented Partitioning Methods

This study adopted the SNIC segmentation method, a streamlined super-pixel image segmentation algorithm [50]. SNIC segmentation divides the image into relatively uniform small regions, reducing the computational complexity of subsequent analyses [51]. Additionally, it combines pixels’ space and color information for segmentation, yielding more accurate results while preserving image details and edge information, and minimizing over-segmentation issues [52]. The SNIC segmentation was performed using built-in functions on the GEE platform. After experimentation and optimization, the super-pixel size was set to 50, the compactness parameter to 0.5 to achieve irregularly shaped super-pixels, the connectivity parameter to 8 to specify the connection method between super-pixels, and the neighborhood size to 128 to avoid tiling boundary artifacts [53].

### 3.3. Feature Selection Method

Given the potential for multicollinearity among selected feature variables [22], we employed Pearson correlation analysis to evaluate the correlations between feature variables and ensure the removal of any highly correlated features before applying RFE [54]. The Pearson correlation coefficient (r) quantifies the strength and direction of a linear relationship between two variables, with values ranging from −1 to 1. The formula for calculating the correlation coefficient is as follows:

(1) $$r=\frac{\mathrm{cov}\left(X,Y\right)}{\sigma_{X}\sigma_{Y}}$$

Here, cov(*X*,*Y*) represents the covariance between variables *X* and *Y*, and $\sigma_{X}$ and $\sigma_{Y}$ denote the standard deviations of *X* and *Y*, respectively. The covariance is calculated using the formula:

(2) $$\mathrm{cov}\left(X,Y\right)=\frac{1}{n-1}\sum_{i=1}^{n}\left(X_{i}-\bar{X}\right)\left(Y_{i}-\bar{Y}\right)$$

In this equation, $\bar{X}$ and $\bar{Y}$ are the sample means of variables *X* and *Y*, and *n* represents the sample size.

Recursion feature elimination (RFE) reduces model complexity and the risk of overfitting by eliminating redundant features, thereby enhancing predictive performance [55]. Its implementation involves training an initial model with training data in the GEE platform and calculating importance scores for each feature variable. Feature variables are ranked based on these importance scores, and the lowest-ranked features are removed. The remaining feature variables are then used for the next round of classification. This process continues until the number of feature variables reaches a target amount or until all features have been removed. The result is a refined set of feature variables used for classification.

### 3.4. Random Forest

RF enhances model diversity by using bootstrap sampling to randomly select samples from the original dataset. The selected samples are then used to construct the training set for each decision tree [56]. For each decision tree, the corresponding out-of-bag data are used to calculate the out-of-bag (OOB) error and the variable importance. During the construction process, a subset of features is randomly chosen, reducing feature correlation and increasing model independence [57]. RF integrates multiple decision trees (DTs), and the final prediction is generated by aggregating the predictions from all of the trees. This method effectively reduces overfitting and is robust against noise and outliers [58]. In our study, we constructed a random forest (RF) model with 100 decision trees on the GEE platform. The number of decision trees significantly influences the model’s classification performance. The number of variables for each split was set to five through continuous experimentation, which was a crucial factor in enhancing model performance. We set up 6 schemes based on different feature variables from both pixel and object perspectives to analyze the variations in wetland monitoring, with the goal of improving classification accuracy. Table 2 shows the combination of image-based and object-based schemes.

### 3.5. Precision Evaluation

The classification performances of different models were evaluated using a confusion matrix based on ground-truth sample data. The evaluation metrics included “Overall Accuracy” and “Producer’s Accuracy” [59,60]. “Overall Accuracy” refers to the proportion of correctly classified objects out of the total number of objects. “Producer’s Accuracy” is the ratio of objects correctly classified into a specific category to the total number of objects in that category, according to the reference data. The calculation formulas are as follows:

(3) $$\mathrm{Overall}\;\mathrm{Accuracy}=\frac{\sum n_{i}}{N}\times 100\%$$

(4) $$\mathrm{Producer}’s\;\mathrm{Accuracy}=\frac{A}{B}\times 100\%$$

In Formula (3), $\sum n_{i}$ represents the sum of correctly classified instances across all categories in the confusion matrix, while *N* denotes the total number of classified objects. In Formula (4), *A* represents the number of correctly classified instances within a specific category, and *B* is the total number of instances in that category.

## 4. Results

### 4.1. Feature Variable Selection

**Figure 3:**
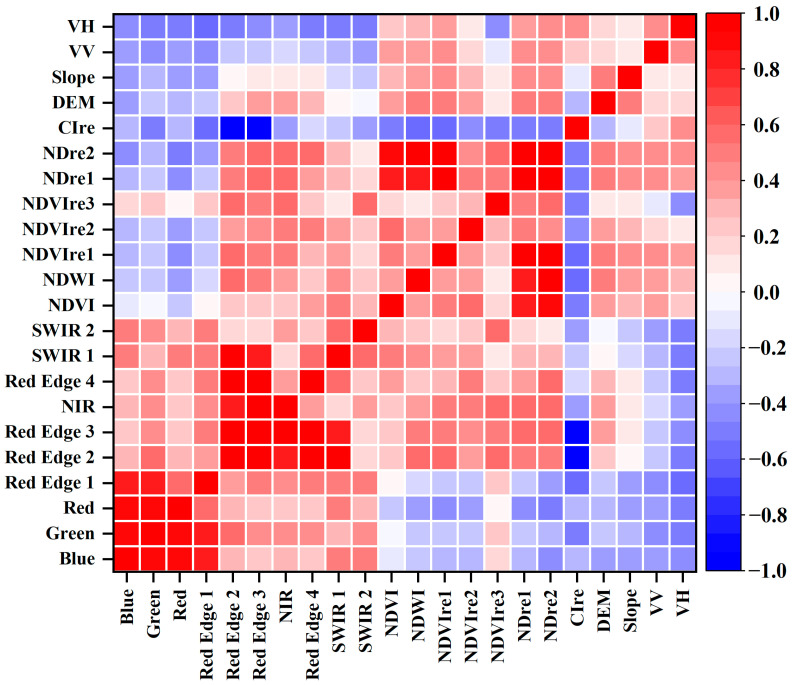
Correlation analysis of characteristic variables.

Figure 3 displays the correlation analysis results between various feature variables. Shades of red indicate a more positive correlation, and shades of blue indicate a more negative correlation, with the intensity of the color representing the strength of the correlation. A correlation coefficient greater than 0.8 is considered a strong correlation. The analysis revealed strong correlations between Blue and Green, as well as Red and Red Edge 1. Similarly, Green was highly correlated with Red and Red Edge 1. Red Edge 2 showed strong correlations with Red Edge 3, NIR, Red Edge 4, SWIR 1, and CIre, while Red Edge 3 strongly correlated with NIR, Red Edge 4, SWIR 1, and CIre. Additionally, NDre1 was strongly correlated with NDVI, NDWI, and NDVIre1, and NDre2 exhibited similar strong correlations with these indices. Due to the relatively close wavelengths and similar spectral characteristics, there was strong correlation between the visible bands and the red-edge bands. To reduce multicollinearity, the highly positively correlated bands Blue, Green, Red Edge 2, and Red Edge 3 were excluded. The red-edge indices were constructed based on red-edge bands, utilizing similar spectral bands in their calculation, leading to similar trends when monitoring vegetation or water bodies and resulting in correlation. Therefore, the highly positively correlated indices NDre1 and NDre2 were excluded from the feature variables.

**Figure 4:**
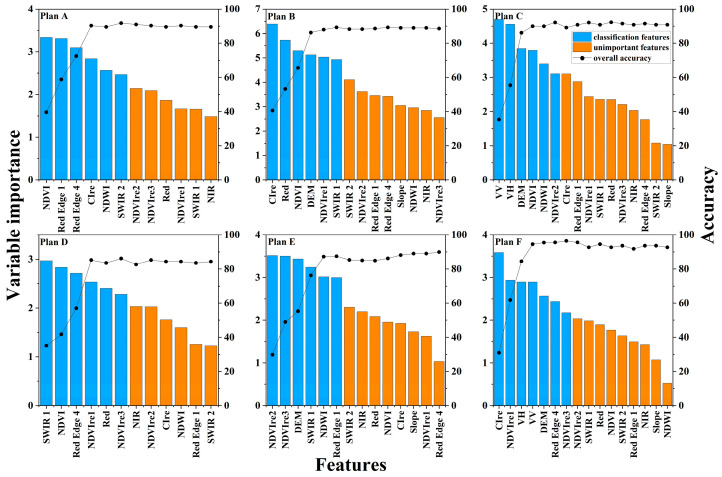
The variable importance measures in descending order and the average overall accuracy of different feature combinations of each Plan.

After excluding highly correlated feature variables, the recursive feature elimination (RFE) method was employed to rank feature importance and remove redundant features. The results indicated that classification accuracy improved as the number of features included in the model increased. However, after incorporating the six or seven features with the highest variable importance, which significantly enhanced the classification accuracy, the further addition of features resulted limited improvements to accuracy. Figure 4 shows that the spectral features Red Edge 4 and SWIR 1, spectral indices NDVI, NDWI, NDVIre1, NDVIre3, and CIre, elevation from terrain features, and radar features frequently appeared as important variables among the six schemes.

### 4.2. Pixel-Based Land Cover Information Extraction Results

**Figure 5:**
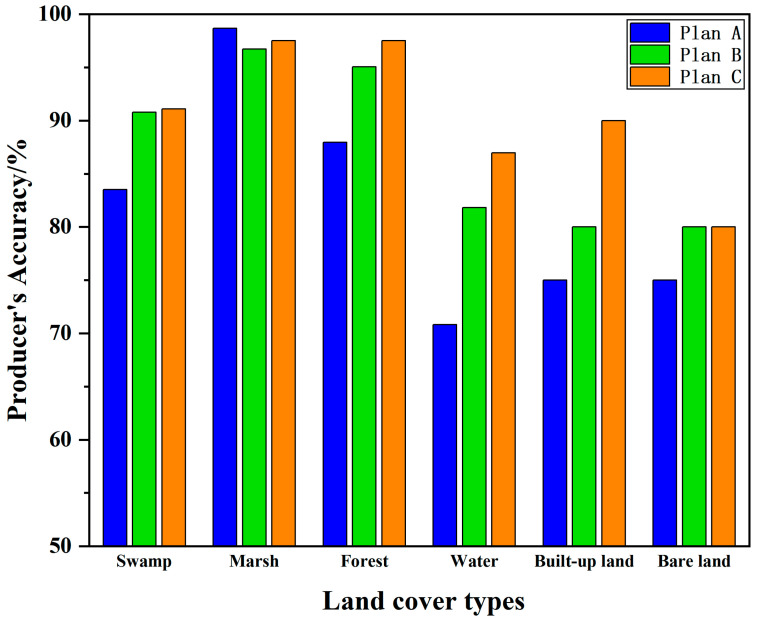
Pixel-based feature classification accuracy.

For the pixel-based schemes, Plan A, Plan B, and Plan C achieved overall accuracies of 86.29%, 91.61%, and 93.55%, respectively. Notably, the accuracy of Plan C increased by 7.26% when terrain and radar features were incorporated. Figure 5 shows that, among the three pixel-based schemes, the classification accuracy of marshes did not improve with the addition of terrain and radar data (see Plans B and C). Instead, the use of optical data was most effective for extracting marsh-related information (Plan A). For other land cover types, the inclusion of terrain data (Plan B) improved classification accuracy, especially for water bodies and forest cover. The combination of optical data and terrain features facilitated the extraction of information for swamps, forests, water bodies, built-up land, and bare land, with the best results for built-up land achieved through this combination. When radar data were further added (Plan C), the combination of optical data, terrain features, and radar features further enhanced the information extraction process for most types (aside from marshes and bare land). This combination significantly improved the classification accuracy for water bodies and built-up land. However, the improvement for other types was not as pronounced compared to Plan B.

**Figure 6:**
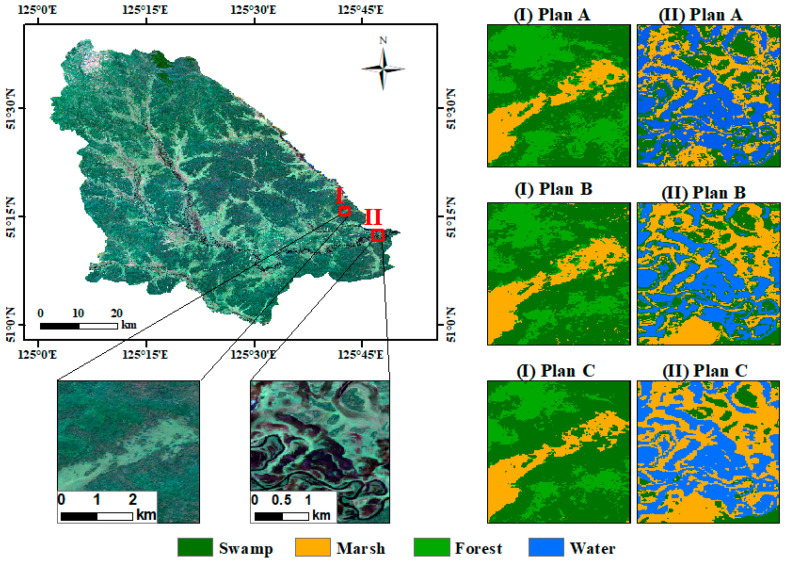
Comparison of classification results based on pixel schemes.

Figure 6 presents a detailed comparison of the results from the three pixel-based schemes. In Plans A and B, the patches of swamps, marshes, and forests were relatively fragmented, leading to a noticeable “salt-and-pepper” effect and some misclassification of swamps and forests. By contrast, Plan C markedly reduced misclassification and the “salt-and-pepper” effect. Marshes were depicted more uniformly and were predominantly found around the edges of swamps in flat terrain, offering a closer alignment with their actual distribution.

### 4.3. Object-Based Land Cover Information Extraction Results

**Figure 7:**
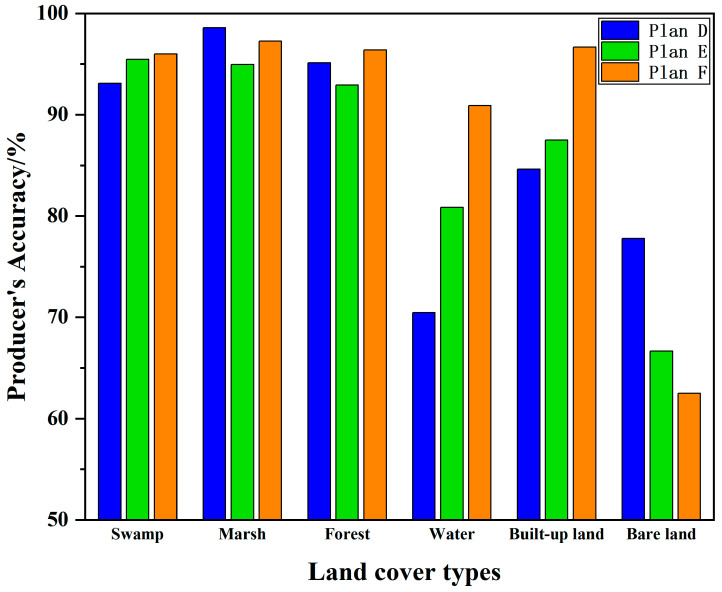
Object-based feature classification accuracy.

For the object-based schemes, Plans D, E, and F achieved overall accuracies of 91.25%, 92.23%, and 95.59%, respectively. Notably, the accuracy of Plan F improved by 4.34% when terrain and radar features were integrated. As shown in Figure 7, among the three object-based schemes, the classification accuracies for swamps, water bodies, and built-up land improved with the inclusion of terrain and radar data (Plans E and F). The combination of optical features, topographic features, and radar features was most effective for extracting information on swamps, water bodies, and built-up land (Plan F), and using this combined approach also yielded optimal results for forest extraction. However, the classification accuracies for marshes, forests, and bare land did not improve with the addition of topographical data (Plan E). Optical data alone were more effective for extracting information on marshes and bare land (Plan D). With the addition of radar data (Plan F), the accuracies for marshes and forests increased, while the accuracy for bare land decreased.

**Figure 8:**
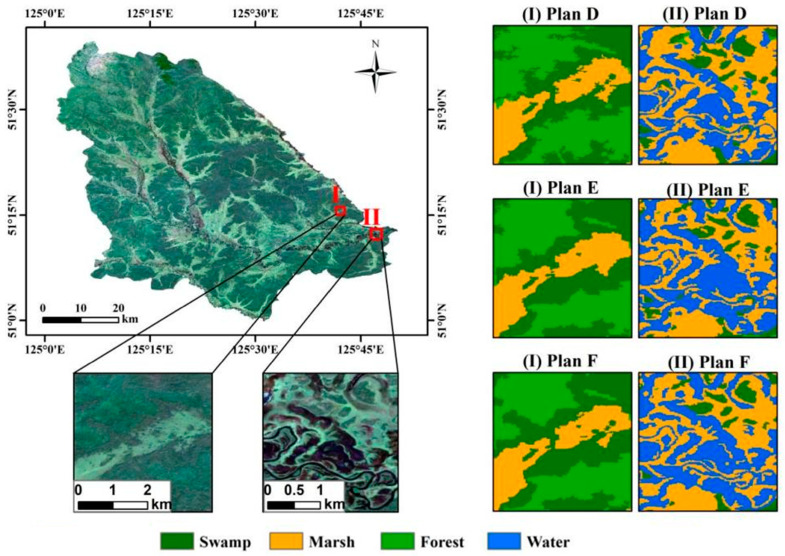
Comparison of classification results based on object schemes.

Figure 8 illustrates a meticulous comparison of the classification outcomes derived from the three object-based schemes. In Plans D and E, inaccuracies occurred where some swamps were misidentified as forests, and some marshes were incorrectly classified as water bodies. However, Plan F rectified these misclassifications, resulting in more defined wetland perimeters and a more cohesive representation of marshes and water bodies. The resulting depiction of Plan F matched the actual conditions, reflecting a noticeable enhancement in the extraction process.

### 4.4. Comparative Analysis of Pixel-Based and Object-Based Classification Results

Based on the figures above, object-based classification modes were clearly more effective for identifying swamps, water bodies, and built-up land, achieving classification accuracies of 96.00%, 90.91%, and 96.67%, respectively. This significantly exceeded the performance of pixel-based classification modes. Conversely, the pixel-based modes yielded higher extraction accuracies for marshes (98.67%), forests (97.53%), and bare land (80.00%). However, these differences in accuracy were not substantial. The object-based schemes demonstrated superior classification accuracy compared to the pixel-based schemes. Additionally, the “salt-and-pepper” effect, an issue found in pixel-based methods, was effectively reduced by the object-based method. Therefore, for the NWR study area, the object-based classification methods proved more suitable.

## 5. Discussion

### 5.1. Comparative Analysis with Available Public Data

**Figure 9:**
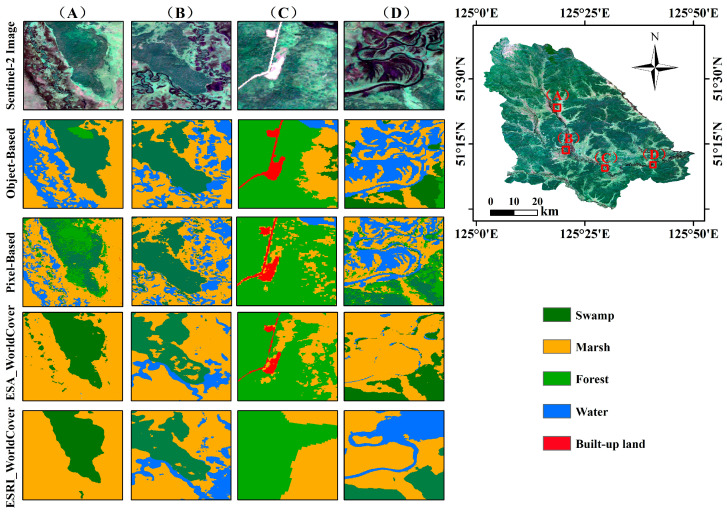
Comparison with other wetland maps (ESA_WorldCover and ESRI_WorldCover). (**A**–**D**) represent four different classification demonstration areas.

This research used high-resolution, freely available Sentinel-1/2 and terrain data to demonstrate the potential of integrating multi-source remote sensing for wetland classification [61]. The wetland information extraction results from this study were compared with other publicly available wetland datasets (Figure 9). The public datasets used include the European Space Agency‘s (ESA) 2020 10-m Global Land Cover Datasets, known as ESA_WorldCover (https://viewer.esa-worldcover.org/worldcover, accessed on 3 June 2024) [62], the Environmental Systems Research Institute’s (ESRI) 2020 10-m dataset, and ESRI_WorldCover, accessible on https://livingatlas.arcgis.com (accessed on 3 June 2024) [63]. The outcomes demonstrated that ESA_WorldCover, which employs a pixel-based classification approach, encountered substantial interference from the “salt-and-pepper” effect during the extraction of forest and built-up land data (Figure 9C). Furthermore, the dataset’s capacity for precise water body delineation was suboptimal, as evidenced by Figure 9A,B,D. ESRI_WorldCover exhibited unclear boundaries and misclassification for swamps, marshes, and forests. Additionally, it inadequately captured water bodies and built-up land, as illustrated in Figure 9C,D. In contrast to the existing datasets, the integration of multi-source remote sensing data significantly enhanced overall classification accuracy. Pixel-based approaches demonstrated particular advantages in classifying marshes, effectively capturing the intricate variability of these environments. Additionally, this method showed strength in accurately identifying built-up land, achieving favorable classification results (Figure 9C). Conversely, the object-based classification approach exhibited greater advantages for swamps (Figure 9B). This method capitalizes on the contextual relationships between neighboring pixels, allowing for a more nuanced understanding of complex swamps. By enhancing the delineation of forested areas, the object-based approach improves the accuracy of classifications related to swamps. The object-based approach, utilizing a combination of optical data, terrain features, and radar characteristics, effectively addressed the challenges associated with extracting fragmented and diminutive water bodies (Figure 9D), distinctly delineated the boundaries of marshes (Figure 9B), and substantially enhanced the extraction accuracy for built-up land areas. By fully leveraging the strengths of optical, radar, and terrain data, we maximized their utility in land cover information extraction. Additionally, we were able to efficiently and swiftly extract land cover information for the NWR by combining the SNIC scale segmentation method with the GEE platform. Despite the success of this research in utilizing multi-source remote sensing data to delineate swamp and forest information across low-mountain and hilly regions, pinpointing the boundaries between mixed swamp and forest zones remains a formidable challenge. This difficulty primarily arises from the canopy cover, which obscures the boundaries between swamps and forests.

### 5.2. Categorical Uncertainty Analysis

The uncertainty in wetland information extraction has been influenced by factors such as image quality [64,65], sample quality, classification algorithms, and land cover classification schemes [66]. In our study, the primary sources of uncertainty were image quality and the distribution and quantity of sample points.

We selected images with minimal cloud cover during the peak vegetation growth season from July to August 2020. However, some regions still experienced significant cloud cover. Despite conducting cloud removal procedures on the GEE platform, there was still an inevitable lack of data completeness and precision, leading to classification uncertainty. Additionally, cloud removal risked partial data loss in single images [67]. While cloud-free images from similar periods were used to fill these gaps, spectral differences between images were found to be unavoidable, unfortunately increasing uncertainty in wetland information extraction.

Regarding sample point selection, discrepancies between the collection time of samples and the acquisition time of remote sensing images also impacted the stability and reliability of wetland monitoring. Wetland type distribution changes over time [68], primarily due to dynamic water fluctuations, which alter wetland boundaries [69]. These changes are more pronounced during the rainy summer months. Therefore, it is crucial to ensure that sample collection times are as consistent or close to the remote sensing image times as possible. Additionally, uneven spatial distribution and insufficient numbers of sample points contributed to uncertainties in wetland monitoring [67]. Choosing representative and sufficiently numerous sample points is an effective way to improve classification accuracy and reduce uncertainty [70].

Research suggests that, for wetland information extraction, relying on images from a specific timeframe cannot fully mitigate the effects of long-term wetland dynamics on extraction results [61]. We intend to integrate long-term time series images and phenological characteristics to create a multi-dimensional feature set for our wetland extraction model. This approach will facilitate the extraction of more detailed information on wetland dynamics, thereby achieving greater accuracy and broader applicability in wetland information extraction results.

## Figures and Tables

**Table 1:** Description of spectral indices and radar variables.

Characteristic Variable	Full Name of the Index	Abbreviation	Characterization or Formula	Reference
Spectral index	Normalized Difference Vegetation Index	NDVI	(NIR − Red)/(NIR + Red)	[39,40]
	Normalized Difference Water Index	NDWI	(Green − NIR)/(Green + NIR)	[41]
	Normalized Difference Vegetation Index red-edge 1	NDVIre1	(NIR − Red Edge 1)/(NIR + Red Edge 1)	[42]
	Normalized Difference Vegetation Index red-edge 2	NDVIre2	(NIR − Red Edge 2)/(NIR + Red Edge 2)	[43]
	Normalized Difference Vegetation Index red-edge 3	NDVIre3	(NIR − Red Edge 3)/(NIR + Red Edge 3)	[43]
	Normalized Difference red-edge 1	NDre1	(Red Edge 2 − Red Edge 1)/(Red Edge 2 + Red Edge 1)	[42]
	Normalized Difference red-edge 2	NDre2	(Red Edge 3 − Red Edge 1)/(Red Edge 3 + Red Edge 1)	[44]
	Chlorophyll Index red-edge	CIre	Red Edge 3/Red Edge 1 − 1	[45]
Topographic features	Digital Elevation Model	DEM	Elevation	[48]
	Slope gradient	Slope	Slope	[48]
Radar features	Vertical–Vertical Backscatter	VV	$\sigma_{VV}^{0}$	[49]
	Vertical–Horizontal Backscatter	VH	$\sigma_{VH}^{0}$	[49]

**Table 2:** Combination of image-based and object-based schemes.

Modes	Schemes	Remote Sensing Features
Pixel-Based	Plan A	Spectral bands and Spectral indices
Pixel-Based	Plan B	Spectral bands, Spectral indices, DEM, and Slope
Pixel-Based	Plan C	Spectral bands, Spectral indices, DEM, Slope, and Radar back (VV, VH)
Object-Based	Plan D	Spectral bands and Spectral indices
Object-Based	Plan E	Spectral bands, Spectral indices, DEM, and Slope
Object-Based	Plan F	Spectral bands, Spectral indices, DEM, Slope, and Radar back (VV, VH)

## Data Availability

The data presented in this study are available on request from the corresponding author.

## Supplementary Materials

The following supporting information can be downloaded at: https://www.mdpi.com/article/10.3390/s24206664/s1, Table S1: Sentinel-2 spectral bands introduction.
