# Supplementary material for: Multi-Source Remote Sensing Data for Wetland Information Extraction: A Case Study of the Nanweng River National Wetland Reserve
Source: Sensors (Basel). 2024 Oct 16;24(20):6664. doi: 10.3390/s24206664 (PMC11511420; doi:10.3390/s24206664)
Supplement: Supplementary file 1 [file sensors-24-06664-s001.zip › sensors-3208765-supplementary.pdf]

Supplementary Materials:

Table S1. Sentinel-2 spectral bands introduction.

Band	Spatial Sample Distance(m)	Central Wavelength(nm)	Bandwidth(nm)
Blue	10	490	65
Green	10	560	35
Red	10	665	30
Red Edge 1	20	705	15
Red Edge 2	20	740	15
Red Edge 3	20	783	20
NIR	10	842	115
Red Edge 4	20	865	20
SWIR 1	20	1610	90
SWIR 2	20	2190	180
